# Feature Selection for Breast Cancer Classification by Integrating Somatic Mutation and Gene Expression

**DOI:** 10.3389/fgene.2021.629946

**Published:** 2021-02-26

**Authors:** Qin Jiang, Min Jin

**Affiliations:** College of Computer Science and Electronic Engineering, Hunan University, Changsha, China

**Keywords:** breast cancer, machine learning, classification, feature selection, gradient boosted decision tree

## Abstract

Exploring the molecular mechanisms of breast cancer is essential for the early prediction, diagnosis, and treatment of cancer patients. The large scale of data obtained from the high-throughput sequencing technology makes it difficult to identify the driver mutations and a minimal optimal set of genes that are critical to the classification of cancer. In this study, we propose a novel method without any prior information to identify mutated genes associated with breast cancer. For the somatic mutation data, it is processed to a mutated matrix, from which the mutation frequency of each gene can be obtained. By setting a reasonable threshold for the mutation frequency, a mutated gene set is filtered from the mutated matrix. For the gene expression data, it is used to generate the gene expression matrix, while the mutated gene set is mapped onto the matrix to construct a co-expression profile. In the stage of feature selection, we propose a staged feature selection algorithm, using fold change, false discovery rate to select differentially expressed genes, mutual information to remove the irrelevant and redundant features, and the embedded method based on gradient boosting decision tree with Bayesian optimization to obtain an optimal model. In the stage of evaluation, we propose a weighted metric to modify the traditional accuracy to solve the sample imbalance problem. We apply the proposed method to The Cancer Genome Atlas breast cancer data and identify a mutated gene set, among which the implicated genes are oncogenes or tumor suppressors previously reported to be associated with carcinogenesis. As a comparison with the integrative network, we also perform the optimal model on the individual gene expression and the gold standard PMA50. The results show that the integrative network outperforms the gene expression and PMA50 in the average of most metrics, which indicate the effectiveness of our proposed method by integrating multiple data sources, and can discover the associated mutated genes in breast cancer.

## Introduction

Breast cancer is considered to be the most prevalent cancer among women and the second common cause of death in both developed and undeveloped countries. It is caused by multiple factors including genomic, transcriptomic, and epigenomic involvement in its formation and development. With the development of technology, understanding the pathogenesis of cancer from the perspective of molecular contributes to effective diagnosis and treatment. The large-scale cancer genomics project, The Cancer Genome Atlas (TCGA) (Tomczak et al., 2015), has produced a large volume of data, providing ways to explore cancer formation and progression.

In general, the cancer transcriptome contains gene expression, including messenger RNA (mRNA), long non-coding RNA (lncRNA), and microRNA (miRNA). Previous studies focused on utilizing the gene expression profile to successfully diagnose individuals based on the differential gene expression (Li et al., 2017) and other clinically relevant phenotypes. Meanwhile, the cancer genome contains many mutations. Among them, one of the most important is somatic mutations, which include single-nucleotide variant (SNVs) and small insertions and deletions (indels). Some mutations that contribute to cancer progression from normal to malignant are called driver mutations, and others that accumulate in cells but do not contribute to cancer development are called passengers (Bozic et al., 2010). Distinguishing driver mutations from the passengers that have no critical effect on cancer cells is a crucial step and challenging task in understanding the molecular mechanisms of cancer, which can guide effective treatment and prognosis for cancer patients and promote the development of targeted drugs. In earlier studies, researchers focused on detecting driver genes that cause tumors (Merid et al., 2014). A common approach is to identify driver genes by detecting positive signals in tumors. Because of the complexity of the cancer genome, driver genes contain not only driver mutations but also passenger mutations. This makes this kind of approach sometimes ineffective.

On the other hand, studies have shown that somatic mutations frequently perturb the expression level of affected genes and thus disrupt the pathways controlling normal growth (Kwong et al., 2020). For example, mRNAs carrying a premature stop codon, which can be introduced by truncation mutations, are typically eliminated by the process called nonsense-mediated mRNA decay, and thus, both the concentration of mRNA transcripts and protein products would be decreased owing to truncation mutations (Jia and Zhao, 2016). Considering the association between the somatic mutation and gene expression, several studies have emphasized the necessity of integrating both types of data to identify candidate driver genes (Masica and Karchin, 2011; Zhang and Wang, 2020). For cancer analysis, many researchers construct a co-expression network by integrating different types of data. He et al. (2017) and Wu et al. (2019) utilized the network by integrating somatic mutation with gene expression to identify the type of cancers and cancer subtypes. Mamidi et al. (2019) integrated germline and somatic mutation to discover biomarkers in triple-negative breast cancer and identified the molecular networks and biological pathways.

As the molecular network has been verified to be effective for the biological discovery of cancers, current studies utilized the network across different types of cancer or cancer subtypes. However, the objective of most researches is the universality of the methods, which makes it difficult to be equally effective in all disease types. In this study, we aim to construct an efficient method of architecture for the diagnosis of breast cancer based on the network of somatic mutation and gene expression. We are focused not only on finding more biomarkers but also on the classification performance of the model. First, the somatic mutation is used to generate a binary mutation network; similarly, an expression network is obtained from the gene expression profiles. Then, for the expression network, we compute both the observed *p*-value and the adjusted *p*-value to correct for multiple-hypothesis testing (false discovery rate, FDR) and thus obtain the differential expression network. Meanwhile, an integrative network is constructed by combining the mutation network and the differential expression network. Thirdly, we rank the genes in the integrative network by mutual information (MI) and select the top 50 genes, which are highly correlated with breast cancer. Finally, we use the Bayesian optimization method to optimize the classification model, gradient boosting decision tree (GBDT), which is further applied to assess the features selected from the previous step. In terms of evaluation metrics, the traditional metric of accuracy does not consider the sample imbalance, so we propose a simple and effective metric, balanced accuracy, to reveal the ability of the different model to classify positive and negative samples.

## Materials and Methods

**FIGURE 1:**
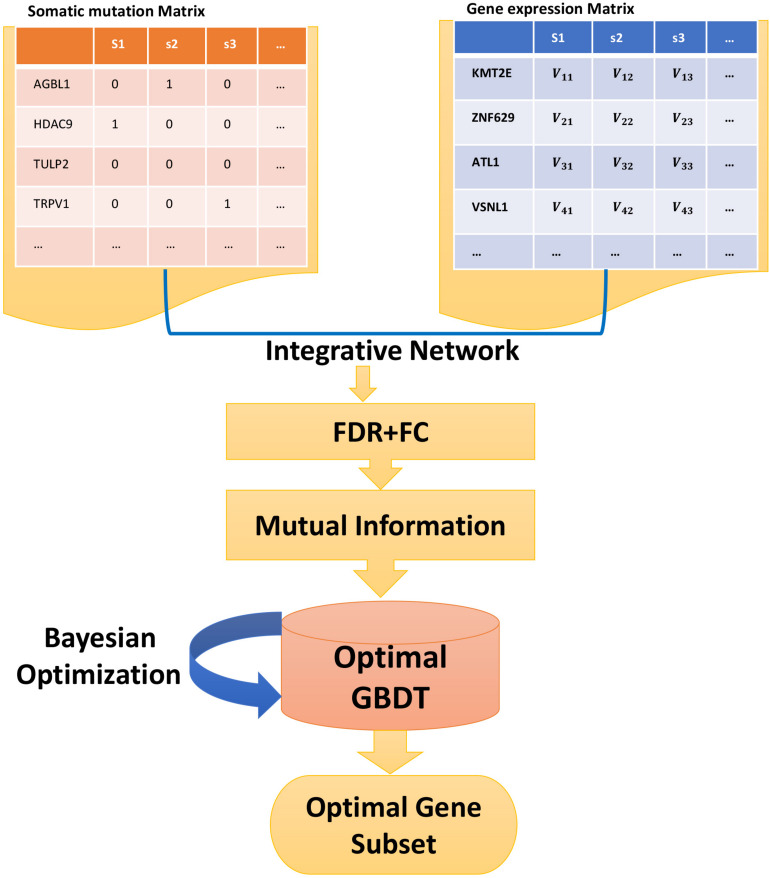
The flowchart of the proposed method. From the somatic mutation matrix, the mutation frequency of each gene is obtained to select the highly mutated genes, which will be integrated by mapping into the gene expression matrix to get the integrative network. After FDR, FC, and mutual information ranking, the feature genes serve as the input to GBDT. Then, the optimal model is obtained by Bayesian optimization. During the training process, the optimal gene subset is obtained simultaneously.

We used statistical and machine learning methods to develop this novel method for feature selection and classification, including the preprocessing of data, filter method, and embedded method for feature selection, processing of imbalanced data, and the final classification model. Figure 1 shows the flowchart of the proposed method.

### Dataset Construction and Preprocessing

In this research, we use publicly available breast cancer datasets (BRCA) from TCGA, including transcriptome gene expression and somatic mutation. Considering the different structures of these two types of data, we used different methods to preprocess them. Table 1 shows the numbers of samples and features for the two datasets.

**TABLE 1:** Confusion matrix for statistical tests.

	H_0_ is true	H_1_ is true	Total
Significant	V	S	R
Not significant	U	T	m-R
Total	m_0_	m-m_0_	m

*H_0_ is the null hypothesis, H_1_ is the alternative hypothesis or reject null hypothesis. m is the number of hypothesis tests. m_0_ is the number of null hypotheses that are true. m-m_0_ is the number of alternative hypothesis that are true. V is the number of false-positive cases. S is the number of the true positive cases. U is the number of true negative cases. T is the number of false negative cases. R = V + S is the number of rejected hypotheses. FDR = E(V/R).*

The BRCA gene expression dataset comprises 1222 samples and 57,063 genes. There are 113 normal samples and 1109 tumor samples. We used the edgeR package to filter the genes expressed in small amounts in most samples and normalized the data. The gene expression data was reduced from 57,063 to 34,465 by deleting the genes expressed in small amounts in most samples.

The somatic mutation data comes from the simple nucleotide variation (SNV) in the TCGA-BRCA project. The data file includes SNP, INS, and DEL, three types of mutations. The important fields in the data file are Hugo_Symbol (gene name), Variant_Type, and Tumor_Sample_Barcode (sample name). Statistically, the somatic mutation data contains 18,127 genes and 986 samples. To get the mutation frequency of each gene in all samples, we use a Perl script to process the data file. For example, if gene A is present in sample S, that means sample S has a mutation in gene A, then we code it as “1,” otherwise we code it as “0.” Supplementary Table 1 shows the coding schedule of all genes in samples. Given the sample set S = {s_1_, s_2_, …s_*n*_}, n is the total number of samples, and s_*i*_ represents the sample i. Gene set G = {g_1_, g_2_, …g_*m*_}, m is the total number of mutation genes, and g_*j*_ represents the gene j. In the set of sample number C = {c_1_, c_2_, …c_*m*_}, c_*k*_ represents the number of samples with “1” in gene k. The set C can be calculated by the number of “1” in each row in Supplementary Table 1.

According to Supplementary Table 1 and set C, we can obtain the frequency of mutations across patients to assess the percentage of patients carrying a particular mutation in each mutated gene. To further reduce the interference of genes with low mutation rates, we set the threshold p as the percentage of the total samples to select the genes with high mutation frequency. The selected gene set constitutes the mutation network. In the experiment, we compare the effects of different p on classification accuracy by the proposed model, and the result is shown in Supplementary Table 2. Due to the highest accuracy 97.31% obtained by setting the threshold p as 1%, we apply this value in the proposed method.

### The Way to Combine Somatic Mutation and Gene Expression

Somatic mutations in cancer genomes frequently perturb the expression level of affected genes. Then, the pathways controlling normal growth are disrupted (Zhang et al., 2013). Similarly, the research by Ding et al. (2015) assessed the impact of mutations on gene expression as a means of quantifying potential phenotypic effects and for novel cancer gene discovery. Fleck et al. (2016) addressed the issue of cancer heterogeneity by using both somatic mutation and gene expression data and proposed a formulation to model the molecular progression of cancer. They discovered that the progression of the disease was reflected in both the accumulation of mutations and changes in gene expression levels. Further study (Jia and Zhao, 2016) focused on the functional footprints of somatic mutations in 12 cancer types and grouped the mutations by mutation type, cluster, and status. This study unraveled the effects of somatic mutation features on mRNA and protein expression.

Our study is based on the assumption that mutations may cause changes in the cell’s state, such as underexpression or overexpression of different genes. Then, we combine the somatic mutation network with the gene expression network to obtain an integrative network. In the integrative process of the two types of networks, we refer to the gene expression network to obtain the expression value of the somatic mutation genes in the mutation network. It is important to note that in the subsequent classification task, the normal samples in the expression network are added as the control group.

### Fold Change and False Discovery Rate

Fold change (FC) is used to calculate the differential multiples of gene expression values between cancer samples and normal samples, which is the basic method for detecting differential genes, and represents the expression values of feature *i* and sample *j* in cancer samples and normal samples; FC is defined as:

(1) $$FC_{i}=\frac{{\bar{X}}_{i}}{{\bar{Y}}_{i}}.$$

When FC exceeds the initial set threshold, it can be considered that the feature is different, and it is generally considered that there is a significant difference when the difference multiple is more than 2. FC can directly obtain the differentially expressed values, but in the absence of false-positive control, the rate of false-positive results is relatively high.

According to statistical theory, in multiple-hypothesis testing, it is important to control the probability of making mistakes in multiple statistical inferences, called FDR. FDR can be used to analyze deferentially expressed genes to control the proportion of false positives (Reiner-Benaim, 2010). Table 2 shows the confusion matrix for the statistical test. FDR can be defined as follows:

(2) $$FDR=E\left(\frac{V}{V+S}\right)=E\left(\frac{V}{R}\right)\left(R>0\right).$$

**TABLE 2:** The optimal parameters for each step in the proposed method.

Parameter	p	FDR	| log(FC)|	*M*
**Threshold**	1%	0.05	1	50

*p is the percentage of the total samples, which represents the mutation frequency of a certain gene. FDR is the false discovery rate, and FC is the fold change. M is the number of genes that top ranking in mutual information.*

The number of false positives in multiple-hypothesis tests can be controlled by controlling that FDR is below the threshold *q*. In general, keep FDR below 0.01, or ensure that there is at most one false positive for every 100 positive hypotheses. Feature genes with significant differences can be identified by FC and FDR, but these two methods do not evaluate the classification performance of these features.

Fold change and FDR are applied to integrative data to select the differentially expressed genes. By comparing the classification balanced accuracy under different FC and FDR thresholds shown in Supplementary Tables 3, 4, the optimal value of FC and FDR thresholds is obtained: log (FC) > 1.0, FDR < 0.05.

### Mutual Information

Mutual information (Bonev et al., 2008) is a useful measure of information in information theory and is a kind of filter method. It refers to the correlation between two events set. The datasets consist of tens of thousands of gene columns and one label column. The gene column is defined as *G*_*i*_, and the label column is defined as L. *MI*(*G*_*i*_,*L*) is represented as the MI between the gene *G*_*i*_ and the label L. The calculation equation is Eq. 3.

(3) $$MI\left(G_{i},L\right)=H\left(G_{i}\right)+H\left(L\right)-H\left(G_{i},L\right)$$

*H*(*G*_*i*_) is the information entropy of the gene column *G*_*i*_, *H*(*L*) is the information entropy of the label L, and *H*(*G*_*i*_,*L*) is the joint information entropy of *G*_*i*_ and L. According to information theory, the information entropy is a measure of the uncertainty of a random variable. Suppose X is a random variable, and the range of possible values is *S_x_*, *x* ∈ *S*_*x*_ and the probability is *p(x)*; the information entropy of X is defined as:

(4) $$H\left(X\right)-\sum_{x\in S_{x}}p\left(x\right)logp\left(x\right)$$

*H*(*X*,*Y*) is the joint information entropy, defined as:

(5) $$H\left(X,Y\right)-\sum_{x\in S_{x}}\sum_{y\in S_{y}}p\left(x,y\right)logp\left(x,y\right)$$

*p(x, y)* is the joint probability density function. *MI*(*G*_*i*_,*L*) can be calculated according to Eqs 4 and 5. In our study, MI is used to measure the dependency between a feature and the classification type. In general, the greater value of MI indicates that the feature contains more information for classification. Therefore, we rank the MI values of each feature and selected the top M features from the integrative data, respectively. The final objective of this method is to remove irrelevant features to reduce the dimension of integrative data. We set different values of M to compare the classification-balanced accuracy and obtain the best value of M. The result in Supplementary Table 5 shows that the optimal M is 50. Table 3 shows the main parameters applied in the proposed method.

**TABLE 3:** Classification accuracy and balanced accuracy of proposed method.

Case	Testing accuracy	Testing balanced accuracy	Running time
1	0.9796	0.8547	65.2642
2	0.9878	0.9111	20.7672
3	0.9878	0.9255	0.2187
4	**0.9951**	**0.9731**	**0.1925**

*The method in case 1 without using any feature selection and the accuracy is the lowest and is time-consuming. In case 2, using FC + FDR to select differentially expressed genes, the results are improved by 0.84 and 6.6%. In case 3, using FC + FDR and MI to select the key 50 features, 1.58% improvement in the balanced accuracy and a significant reduction in running time are obtained. The proposed method shown in case 4, the best performance in the three metrics is obtained. The bold values are the best results.*

### GBDT With Bayesian Optimization

The filter methods obtain a feature subset for which the discriminative capability is limited for classification purposes. Embedded methods can be used to search the optimal feature subset by a given classifier. In the training procedure, the features with high importance can be selected by ranking and the classification algorithm is optimized simultaneously. It is helpful to build a strong link between the feature subset and the classifier. The GBDT is an ensemble learning algorithm based on GBM, which is proposed by Friedman (Friedman, 2001). During the training process, multiple iterations are used to build multiple trees to make joint decisions. When the square error loss function is adopted, each regression tree learns the conclusions and residuals of all previous trees, and a current residual regression tree is obtained by the fitting. The meaning of residuals is as follows:

residuals=truevalue-predictvalue

The boosting tree (Galicia et al., 2018) is an accumulation of regression trees generated during the entire iteration process. The optimization process of learning is realized by using an additive model and a forward step algorithm. The GBDT was used in our study because of its flexibility for different types of data, excellent classification performance, and robustness for abnormal values.

However, it is tedious and important work to tune the hyperparameters when conducting the GBDT, because it greatly affects the performance of the algorithm. Manual tuning is time-consuming; grid and random searches (Bhat et al., 2018) require no human effort but a long-running time. Therefore, in this research, Bayesian optimization is adopted to find the optimal hyperparameters, which is first proposed by Snoek et al. (2012). Bayesian optimization seeks to minimize the value of the objective function by establishing an alternative function based on the objective function’s past evaluation results. The Bayesian method is different from random or grid searches as they consider previous estimates when testing the next set of hyperparameters, thus saving a lot of effort.

Suppose hyperparameters set (represents a hyperparameter’s value), the relationship between this set, and the loss function that need to be optimized, defined as *f*(*X*). However, machine learning just likes a black box, which means we only know the input and output; *f* is hard to be sure. So we should turn our attention to a function that can be solved. Assume function, we need to find in:

(6) $$x^{*}=argmin_{x\in X}f\left(x\right)$$

Here, we chose Hyperopt in Python library, which adopted Tree Parzen Estimator (TPE), which used the Gaussian Mixture Model (Oh et al., 2019) to learn hyperparameters. First, we split the integrative dataset into 80% learning set and 20% test set then divided the learning set into 60% training set and 40% validation set. The performance of hyperparameters was evaluated on the validation set. The Bayesian optimization assigned a greater probability to the value of the hyperparameters set with a lower loss in the cross-validation. Finally, the best hyperparameters set was output.

### A Weighted Metric for Imbalanced Dataset

Class imbalance is a situation in which the number of training samples of different categories varies greatly in the classification task. There are many strategies to deal with the imbalance problem, such as undersampling and oversampling. EasyEnsemble is a method of undersampling, proposed by Li and Liu (2014). Multiple different training sets are generated by putting back the samples several times, and then multiple different classifiers are trained. The final result is obtained by combining the results of multiple classifiers. Another method is BalanceCascade (Liu et al., 2009), which adopts the idea of Boosting. It also uses undersampling to generate a training set, but those correctly classified samples are not put back. Undersampling is easy to lose information, and the way the final result is integrated also has an impact. The most common strategy for oversampling is SMOTE (Synthetic Minority Oversampling Technique) (Blagus and Lusa, 2013). In this method, the new samples are synthesized according to the nearest neighbor in the minority samples and then added into the dataset. However, there two main problems in this algorithm: there is some blindness in the selection of the nearest neighbor and the problem of distribution marginalization is easy to occur. Additionally, undersampling and oversampling may change the distribution of data. For the task of cancer classification, the size of sample is small, more than a thousand at most, and these strategies do not seem appropriate. Therefore, in this study, we propose a weighted metric to modify the traditional accuracy metric instead of changing the distribution of the dataset. There are far more cancer samples than normal samples, which will lead to the high accuracy of the learning method if it returns a learning model that always predicts the new sample as a cancer category. To solve this problem, we separated the total sample set into a normal set and tumor set. The classification accuracy of the model in the two-sample space embodies the model’s ability to correctly classify the positive and negative samples, named the weight for the two-sample spaces. On the final test stage, we multiply this weight with the accuracy of two sample spaces on the test set.

Let *N* and *T* denote the sample set of normal class and that of tumor class, respectively. $\vec{w_{n}}$ and $\vec{w_{t}}$ are the accuracy of normal samples and tumor samples of classifier *clf* in the validation set, respectively. These two weights represent the different capacities of the given classifier for different types of samples. In the final testing stage, the optimized GBDT is conducted as the classifier to predict the independent test set; $\vec{w_{n}}$ and $\vec{w_{t}}$ will be considered in the final decision. As we split the dataset into 10 equal-sized datasets, $\vec{w_{n}}$ and $\vec{w_{t}}$ are the average accuracy of the 10 validation sets. Here, the average accuracy of normal samples and tumor samples on the 10 test sets are represented by *acc*_*n*_ and *acc*_*t*_. So the final balanced accuracy is defined as:

(7) $$balancedacc=acc_{n}\cdot {\vec{w}}_{n}+acc_{t}\cdot {\vec{w}}_{t}$$

**FIGURE 2:**
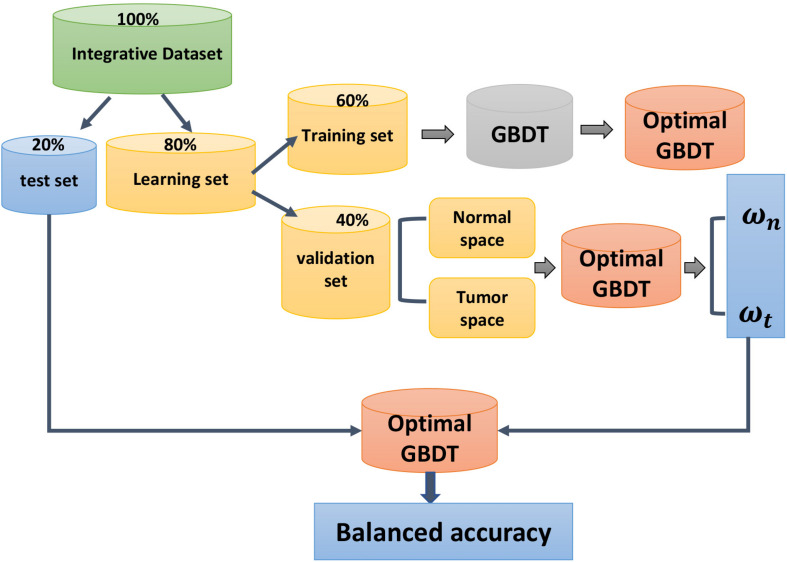
The calculation procedure of balanced accuracy. The raw dataset is split into 10 equal datasets. The diagram shows the procedure on one of the 10 datasets. First, the integrative dataset is derived into a learning set and an independent test set. The learning set is derived into a training set and a validation set. The training set is used to train the GBDT model and the validation set is used to obtain the weight for normal and tumor space (ω_*n*_ and ω_*t*_), which is represented by the accuracy of normal and tumor space. Finally, when the optimal model is tested on the test set, ω_*n*_ and ω_*t*_ will be used to modify the final accuracy to obtain the balanced accuracy.

The core procedure of calculating the weighted metric for balanced accuracy is described in Figure 2. The weighted metric for the imbalanced dataset is easy to operate. It considers the classification ability of the classifier on samples of different categories and further revises the final test results by multiply weights, thus reducing the impact of class imbalance.

### Evaluation Criteria

The following metrics are used to evaluate the performance of the classification model in this study:

Accuracy: $ACC=\frac{TP+TN}{TP+FP+TN+FN}$

Sensitivity: $SES=\frac{TP}{TP+FN}$

Specificity: $SPC=\frac{TN}{TN+FP}$

Precision: $PRC=\frac{TP}{TP+FP}$

_*F*_1__ score: $F_{1}=\frac{2TP}{2TP+FP+FN}$

In this study, the tumor sample is positive, and the normal sample is the negative sample, where TP (true positive) is the number of tumor samples predicted as tumor, FP (false positive) is the number of tumor samples predicted as normal, TN (true negative) is the number of tumor samples normal and predicted as normal, and FN (false negative) is the number of normal samples and predicted as tumor. Meanwhile, the AUC is obtained.

Due to that the number of samples is much smaller than that of the features, in this study, first, we split the dataset into 10 equal-sized datasets. Then, we divide the datasets into 80% learning set and 20% test set and ensure that the test set does not participate in any training process (Meng et al., 2020). Finally, the independent test set is used to calculate the above evaluation metrics. This procedure is repeated on the 10 datasets. The average of the results generated on the 10 datasets is used as the final performance of the proposed model on the test set.

## Results

### Classification Results of Proposed Method SFS

In our experiments, the training set is used to train the classifier. The obtained parameters are verified on the validation set. In addition, we calculate $\vec{w_{n}}$ and $\vec{w_{t}}$ (normal samples’ accuracy and tumor samples’ accuracy in the validation set). Moreover, balanced accuracy was calculated by Eq. 6. The proposed method adopts FC, FDR, MI, and GBDT with Bayesian optimization. The parameters are applied as follows:

(1) FC: |*log*(*FC*)| > 1.0

(2) FDR: *FDR* 0.05

(3) MI: select the top 50 features of MI value ranking

(4) Bayesian optimization: tuning the parameters of GBDT with Bayesian optimization using the 50 features to get the optimal model.

These methods are combined in the ways shown in Table 4.

**TABLE 4:** The mean values of seven evaluation metrics obtained from four methods on integrative dataset.

Classifier	B_ACC	ACC	SES	SPC	PRC	F1	AUC
SVM	0.9413	0.9865	0.9910	0.9435	0.9941	0.9926	0.9672
RF	0.9208	0.9902	**0.9968**	0.9261	0.9924	0.9946	0.9615
KNN	0.9480	0.9914	0.9955	0.9522	0.9950	0.9953	0.9738
Proposed	**0.9731**	**0.9951**	0.9964	**0.9826**	**0.9982**	**0.9973**	**0.9895**

*In the experiments, we randomly split the dataset into 10 equal-sized datasets. The mean values of the seven metrics are obtained on the 10 test sets. The proposed method outperforms other methods in balanced accuracy, accuracy, specificity, precision, F1 score, and AUC. The bold values are the best results.*

Case 1: None of the above methods are used.

Case 2: FC and FDR are used to obtain the differentially expressed genes.

Case 3: FC + FDR, MI are used to select informative features.

Case 4: FC + FDR, MI, and Bayesian optimization are adopted to optimize GBDT, and this case is the proposed method.

The testing accuracy is obtained by the classifier GBDT on the independent test set. The results shown in case 1 are the classification accuracy using GBDT without any feature selection. It can be observed that the GBDT without any feature selection obtains a testing accuracy of about 97.96%, but the testing balanced accuracy is only about 85.47%, which implied the learning efficiency of the GBDT without feature selection is not much high. In case 2, although FC and FDR effectively reduce the running time, it does not improve the accuracy significantly, because they ignore the correlation between features. In case 3, we add MI to further select key features, and the results show that there is an improvement (1.58%) in balance accuracy and a significant reduction in running time. In case 4, we use Bayesian optimization to optimize GBDT to obtain the optimal model. According to the results, we conclude that the accuracy and balanced accuracy are improved by 0.74 and 5.14%, which were compared with case 3. Particularly, the proposed method shown in case 4 obtains the highest testing accuracy and balanced accuracy. The performance of testing balanced accuracy is improved by 13.85%, compared with the method in case 1. From the perspective of vertical comparison, the features selected by the proposed method have better classification performance. From the perspective of horizontal comparison, balanced accuracy improves more than traditional accuracy, which indicates that the proposed model shows greater advantages when the sample balance is considered.

### The Hyperparameters of GBDT Adjusted by Bayesian Optimization

**FIGURE 3:**
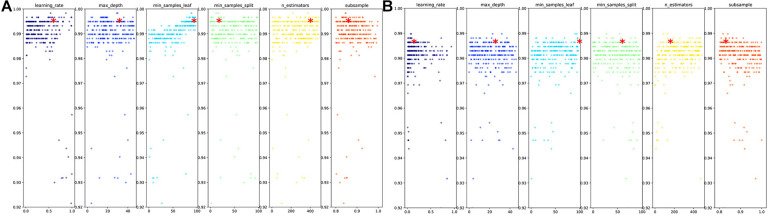
**(A)** Bayesian optimization for hyperparameters of GBDT. The best hyperparameters set: {“learning_rate”: 0.53732209, “max_depth”: 29, “min_samples_leaf”: 88, “min_samples_split”: 12, “n_estimators”: 374, “subsample”: 0.84620375}, testing accuracy: 0.995102041, testing balanced accuracy: 0.973135976. The best hyperparameter set was obtained by comparing the average metrics on 10 test sets. The detailed results obtained by every test are shown in Supplementary Datasheet 1. **(B)** Random search for hyperparameters of GBDT. The best hyperparameters set: {“learning_rate”: 0.0829095, “max_depth”: 23, “min_samples_leaf”: 94, “min_samples_split”: 54, “n_estimators”: 130, “subsample”: 0.817617081}, testing accuracy: 0.994693878, testing balanced accuracy: 0.968032706. The best hyperparameter set was obtained by comparing the average metrics on 10 test sets. The detailed results obtained by every test are shown in Supplementary Datasheet 1.

Bayesian optimization aims to find the minimum value of the objective function by establishing a proxy function (probabilistic model). The proxy function is easier to optimize than the objective function (Victoria and Maragatham, 2020), so the next input value to be evaluated is selected by applying some criterion. For hyperparameter optimization, the objective function is the validation error of the machine learning model using a set of hyperparameters. Its goal is to find the hyperparameters that produce the minimum error on the validation set and to generalize these results to the test set. The cost of evaluating an objective function is significant because it requires the training of a machine learning model with a specific set of hyperparameters. Bayesian hyperparameter tuning uses a constantly updated probabilistic model to “focus” the search process on the hyperparameters that are likely to be optimal by reasoning from past results. In this study, for the objective function, the input was a set of hyperparameters, and the output was the fivefold cross-validation loss with classifier GBDT. We chose Tree Parzen Estimation (TPE) as the optimization algorithm. Figure 3 shows the best sets of hyperparameters obtained by Bayesian optimization and random search with 300 iterations. The balanced accuracy gained on the test set by using the best two sets of hyperparameters in GBDT was 97.31 and 96.8%, respectively. The results indicated that Bayesian optimization outperforms random search in the respect of hyperparameter tuning.

In the comparative experiments, we select three other classifiers, SVM, KNN, and RF. Supplementary Tables 6, 7 and Supplementary Figure 2 show the procedure of tuning parameters for the three classifiers. According to the balanced accuracy obtained in those models, the optimal parameters are as follows:

(1) SVM: C = 1, kernel = “linear”
(2) KNN: n_neighbor = 7, metric = “manhattan”
(3) RF: max_depth: 46, min_sample_leaf: 2, min_sample_split: 94, n_estimators: 75

Table 5 shows the mean values of seven evaluation metrics obtained from four methods on the integrative dataset. The results indicate that the proposed method outperforms SVM, KNN, and RF by 3.4, 5.7, and 2.6% with balanced accuracy. Particularly, the AUC obtained by the proposed method is 2.3, 2.9, and 1.6% higher than the above three classifiers, respectively. We can conclude that the proposed method achieves the best performance on the integrative dataset in terms of balanced accuracy (97.31%), accuracy (99.51%), specificity (98.26%), precision (99.82%), F1 score (99.73%), and AUC (98.95%). Supplementary Datasheet 2 shows the average and variance of each metric, and the proposed method gets the smallest variance in accuracy, balanced accuracy, and F1 score in TCGA-BRCA. Other metrics are the second smallest. It can be seen from the variance table that the proposed method has certain robustness.

**TABLE 5:** Comparison of related works.

Work	Method	Dataset resource	Evaluation metric	Performance
Mavaddat et al., 2019	Polygenic risk scores (PRSs)	Breast Cancer Association Consortium (BCAC)	AUC	0.63
Chaurasia et al., 2018	Naive Bayes	Breast Cancer Wisconsin dataset	Accuracy	97.36%
Ai et al., 2020	Pearson correlation coefficient (PCC) + SVM	GEO	Accuracy	96.92%
Huang et al., 2017	SVM ensembles	UCI and ACM SIGKDD Cup 2008	Accuracy AUC F-measure	96.85% 0.967 0.988

### The Effect of Integrative Dataset

**FIGURE 4:**
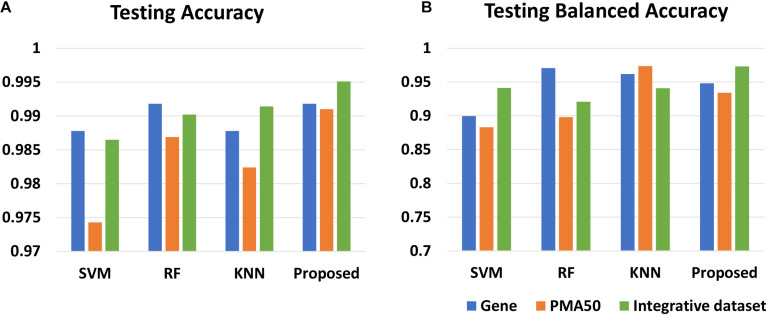
Comparison of the results of different datasets on four classifiers. **(A)** The average testing accuracy obtained by the four methods. **(B)** The average balanced testing accuracy obtained by the four methods. The left data is the accuracy; the height of the blue and red bars represent the performance of each method on gene data and combine data. The red bar obtained by the proposed method in **(A)** is the highest. The red bar obtained by the proposed method in **(B)** is also the highest. That means the proposed method performs best in the accuracy and balanced accuracy on the integrative data.

To explore the effect of the integrative dataset, we apply the proposed method to individual gene expression and integrative dataset, respectively. Besides, we choose PMA50 as the control model. PMA50 refers to a set of 50 genes selected by Parker et al. (2009), which are with a good diagnostic performance that are regarded to be highly related to breast cancer. In Table 6, for the gene expression and PMA50, the proposed method achieves the best testing accuracy. The blue and orange bars in Figures 4A,B intuitively reflect the results. However, for the integrative dataset, the proposed method obtains 99.51% testing accuracy and 97.31% balanced accuracy, which outperforms the gene expression model and PMA50 model. This fact indicates that the features selected by the proposed model have better classification performance.

**TABLE 6:** Comparison between the results of different datasets on four classifiers.

Data category	Testing accuracy				Testing balanced accuracy			
	SVM	RF	KNN	Proposed	SVM	RF	KNN	Proposed
Gene	0.9878	0.9918	0.9878	**0.9918**	0.8995	0.9707	0.9619	0.9481
PMA50	0.9743	0.9869	0.9824	**0.9910**	0.8831	0.8980	0.9736	0.9342
Integrative dataset	0.9865	0.9902	0.9914	**0.9951**	0.9413	0.9208	0.9408	**0.9731**

*For the gene expression, the proposed method obtains the highest accuracy, but the balanced accuracy is highest in RF. For the PMA50, the proposed method obtains the best accuracy. For the integrative dataset, the proposed method obtains the highest accuracy and balanced accuracy, which illustrates that the integrative dataset contains more useful information after feature selection. The bold values are the best results.*

The results in Table 6 and Figure 4 also show the results obtained by the other classifiers. The SVM classifier gives the accuracy of 98.78% on the gene expression dataset, which is higher than that on the integrative dataset. However, the balanced accuracy is higher on the integrative dataset (94.93%). On the other hand, RF and KNN give a higher testing accuracy on the integrative dataset than that on the gene expression dataset, which is illustrated by the blue bars in Figure 4A. However, in Figure 4A, the proposed model obtains the highest three bars, which reveals that the proposed method performs better than other classifiers in all three types of datasets. For a balanced accuracy in Figure 4B, SVM and the proposed model obtain the best results on the integrative dataset, and RF and KNN obtain the best ones on gene expression and PMA50, respectively. The reason for this difference lies in the sensitivity of different classifiers to data distribution. The feature genes in the PMA50 model and the integrative model obtain higher balanced accuracy 97.4% (KNN) and 97.3% (proposed method) than that in the gene expression model, which illustrates that KNN and the proposed method provide the better capability to classify the minority sample class.

### Biomarkers and GO/Pathway Analysis

The 50 genes (listed in the Supplementary Table 8) discovered by the proposed model include 16 genes, *IQGAP3* (Hu et al., 2019), *KIF4A* (Xue et al., 2018), *TSHZ2* (Yamamoto et al., 2011), *MKI67* (Schmidt et al., 2007), *TNXB* (Hu et al., 2009), *KIFC1* (Ogden et al., 2017), *KDM5B* (Catchpole et al., 2011), *PPEF1* (Ye et al., 2020), *RYR3* (Shrestha et al., 2012), *TMEM132C* (Zhang et al., 2020), *FANCD2* (Barroso et al., 2006), *ATAD2* (Kalashnikova et al., 2010), *KIF26B* (Wang et al., 2013), *BRCA2* (Wooster et al., 1995), *BLM* (Arora et al., 2015), and *ARFGEF* (Kim et al., 2011), which are reported to be directly associated with breast cancer by previous researches. Although the other 14 genes have not been verified by biological experiments, we further analyze the Gene Ontology and pathway enrichment to explore their impact on the tumor formation and progression.

**FIGURE 5:**
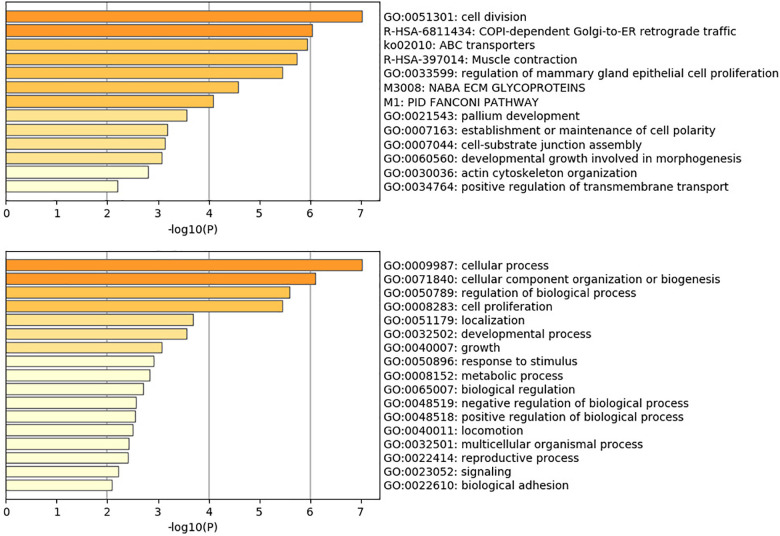
Heatmap of selected Gene Ontology.

Gene Ontology and pathway analysis produces biological function and pathway enriched for mutation genes. The result reveals that *BRCA2*, *KDM5B*, and *IQGAP3* are associated with mammary gland epithelial cell proliferation and gland development; *BLM*, *BRCA2*, *CENPE*, *CENPF*, *KIFC1*, *CKAP*, *CIT*, *TTC28*, *KIF4A*, and *ASPM* are associated with cell division; *BRCA*, *CENPE*, *CENPF*, *FANCD2*, *KIFC1*, *MKI67*, *KIF4A*, and *ASPM* are associated with organelle fission; *BLM*, *BRCA2*, *CENPE*, *CENPF*, *EGFR*, *FANCD2*, *MKI67*, *CKAP5*, and *TTC28* are associated with regulation of the mitotic cell cycle; *ABCA10*, *ABCA9*, *ABCA8*, and *ABCA6* enrich in the pathway of ABC transporters; and *EGFR*, *FN1*, *RELN*, and *TNXB* enrich in the pathway of human papillomavirus infection. The main GO and pathway are shown in Figure 5. The comprehensive analysis of the whole 50 genes is shown in Supplementary Datasheet 3. Overall, the investigation reveals oncogenic interactions and cooperation among mutation genes.

## Discussion

This research presents a Staged Feature Selection method for breast cancer classification based on gene expression and somatic mutation datasets. In the proposed method, FC and FDR were used to select differentially expressed genes, MI was adopted to remove the irrelevant and redundant features, and an embedded method based on GBDT with Bayesian optimization was presented to obtain the informative features. Besides, the weighted metric was proposed to evaluate the classification accuracy, which could avoid the impact of sample imbalance on classification. The experiment results showed that the proposed method selected 50 feature genes and achieved the accuracy of 99.51%, the balanced accuracy of 97.31% and the sensitivity of 99.64%, the specificity of 98.26%, the precision of 99.82%, the F1 score of above 99.73%, and the AUC of 98.95%, which was superior to the other three classifiers. It was verified that the proposed method was an efficient tool for feature selection in breast cancer classification.

The results presented the effectiveness of integration with gene expression and somatic mutation data for breast cancer classification, which indicated that it could provide more useful information for cancer classification by integrating multiple information. However, this study only focused on breast cancer, and the scalability of the proposed method on other types of cancers remained to be further explored, which will provide helpful information for cancer prevention and treatment. Therefore, in future work, we will apply the approach to classify other types of cancer, explore ways to incorporate more relevant data, and introduce other techniques to boost our method. Besides, the pathogenesis of some biomarkers discovered by the proposed model still has to be verified by biological experiments.

## Data Availability Statement

Publicly available datasets were analyzed in this study. This data can be found here: https://portal.gdc.cancer.gov/repository.

## Author Contributions

QJ processed the data, designed the algorithm and the programming codes, and wrote the manuscript. MJ supervised the project and revised the manuscript. Both authors contributed to the article and approved the submitted version.

## Conflict of Interest

The authors declare that the research was conducted in the absence of any commercial or financial relationships that could be construed as a potential conflict of interest.
