# Supplementary figures and images for: Feature Selection for Breast Cancer Classification by Integrating Somatic Mutation and Gene Expression
Source: Front Genet. 2021 Feb 26;12:629946. doi: 10.3389/fgene.2021.629946 (PMC7952975; doi:10.3389/fgene.2021.629946)

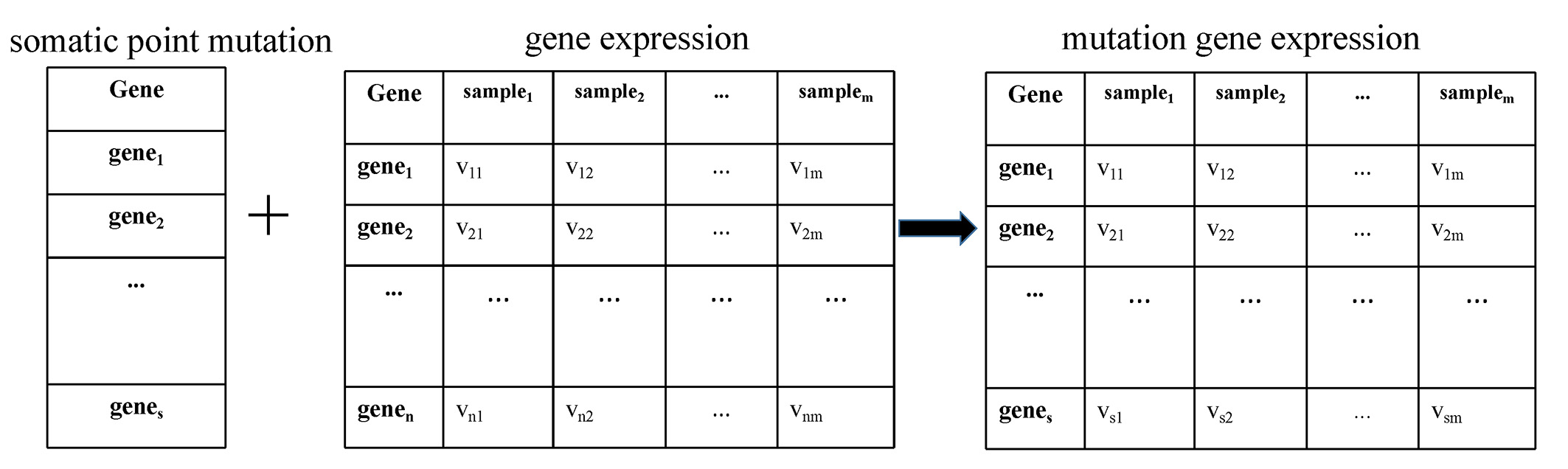

Supplement: Supplementary file 1 [file Image_1.JPEG]

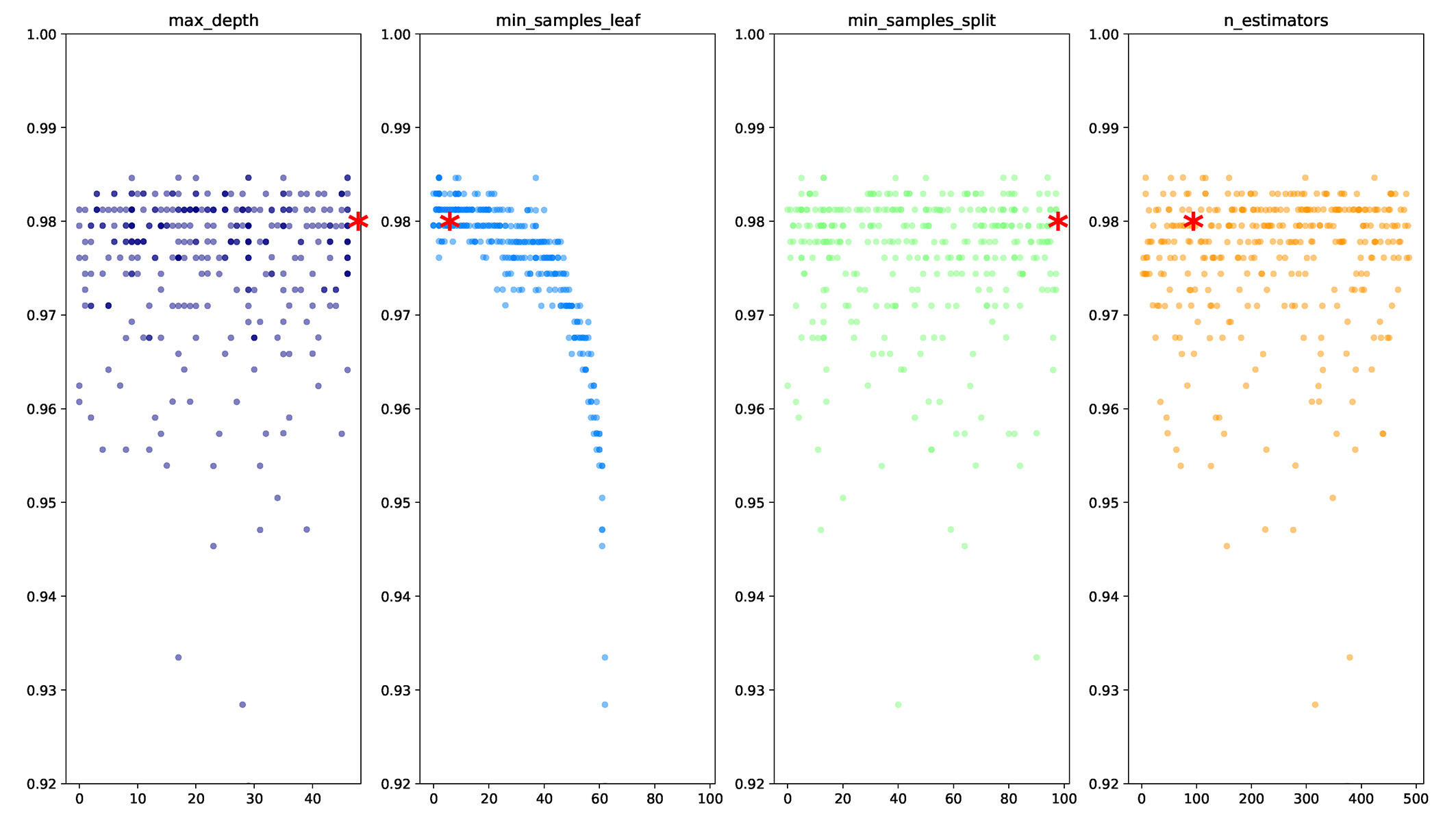

Supplement: Supplementary file 2 [file Image_2.JPEG]
