# Supplementary material for: Feature Selection for Breast Cancer Classification by Integrating Somatic Mutation and Gene Expression
Source: Front Genet. 2021 Feb 26;12:629946. doi: 10.3389/fgene.2021.629946 (PMC7952975; doi:10.3389/fgene.2021.629946)
Supplement: Supplementary file 4 [file Data_Sheet_2.docx]

**Table S1.** Sample distribution of somatic mutation genes

| Gene | TCGA-A2-A3Y0-01A | TCGA-E2-A15S-01A | TCGA-AR-A1AP-01A | … | TCGA-BH-AOAW-01A |
| --- | --- | --- | --- | --- | --- |
| PAG1 | 0 | 0 | 0 | … | 1 |
| PALD1 | 0 | 0 | 0 | … | 0 |
| … | … | … | … | … | … |
| CHAD | 0 | 1 | 0 | … | 0 |

**Table S2.** The relationship between p and the classification accuracy

| p | 0.5% | **1%** | 1.5% | 2% | 2.5% | 3% |
| --- | --- | --- | --- | --- | --- | --- |
| Number of mutation genes | 3872 | **922** | 296 | 123 | 60 | 36 |
| Balanced accuracy | 0.9365 | **0.9731** | 0.9438 | 0.9197 | 0.9458 | 0.9398 |

**Table S3.** The balanced accuracy under different threshold of FDR (p = 1%)

| FDR | <0.01 | <0.05 | <0.1 |
| --- | --- | --- | --- |
| Balanced accuracy | 0.870531 | **0.9731** | 0.924228 |

**Table S4.** The balanced accuracy under different threshold of FC

(p = 1%, FDR<0.05)

| \|log(FC)\| | >1.0 | >1.5 | >2.0 |
| --- | --- | --- | --- |
| Balanced accuracy | **0.9731** | 0.878709 | 0.935317 |

**Table S5.** The balanced accuracy under different value of M

(p=1.0%，FC=1.0, FDR=0.05)

| M | 50 | 100 | 200 | 300 |
| --- | --- | --- | --- | --- |
| Balanced accuracy | **0.9731** | 0.96 | 0.9407 | 0.9141 |

**Table S6.** The procedure of tuning parameters for SVM

| C | 1 | 3 | 5 | 7 | 9 | 11 | 13 |
| --- | --- | --- | --- | --- | --- | --- | --- |
| Balanced accuracy | **0.9413** | **0.9413** | **0.9413** | **0.9413** | **0.9413** | **0.9413** | **0.9413** |

**Table S7.** The procedure of tuning parameters for KNN

| n_neighbor  metric | 3 | 5 | 7 | 9 | 11 | 13 |
| --- | --- | --- | --- | --- | --- | --- |
| minkowski | 0.8665 | 0.8642 | 0.9173 | 0.9004 | 0.9004 | 0.9173 |
| manhattan | 0.9158 | 0.9209 | **0.9408** | 0.9363 | 0.9195 | 0.9195 |

**Table S8.** The 50 gene list of PMA50 and the proposed model

| **PMA50** | **Proposed** |
| --- | --- |
| FOXC1, MIA, KNTC2, CEP55, ANLN, MELK, GPR160, TMEM458, ERBB2, GRB7, FGFR4, BLVRA, BAG1, CDC20, CCNE1, ACTR3B, MYC, SFRP1, KRT14, KRT17, KRT5, MLPH, CCNB1, CDC6, TYMS, UBE2T, RRM2, MMP11, CXXC5, ORC6L, MDM2, KIF2C, PGR, MKI67, BCL2, EGFR, PHGDH, CDH3, NAT1, CDH3, NAT1, SLC39A6, MAPT, UBE2C, PTTG1, EXO1, CENPF, CDCA1, MYBL2, BIRC5 | IQGAP3, KIF4A, ABCA10, CENPF, TSHZ2, ASPM, DMD, ABCA9, MKI67, TNXB, KIFC1, IGSF10, DST, TRPM3, KDM5B, ABCA8, CENPE, SVEP1, PPEF1, GTF3C1, RYR3, DNAH14, TMEM132C, COPA, MYH11, CIT, FANCD2, CKAP5, TTC28, ATAD2, SCN3A, POLQ, EGFR, SORBS1, FREM1, KIF26B, RELN, FN1, PLXNA3, SCN4A, ABCA6, ZNF687 , BRCA2, ALDH1L1, CNTNAP3, BLM, MASP1, WSCD2, PAPPA2, ARFGEF1 |

**Supplementary Figure 1**. The way of combining somatic point mutation with gene expression

**Supplementary Figure 2.** The procedure of tuning parameters for RF

best parameters:

{‘max_depth’:46, ’min_sample_leaf’:2, ’min_sample_split’:94, ’n_estimators’:75}

**Supplementary datasheet 1.** The best hyperparameter sets with Bayesian and Random method for every splitted dataset. Applied the 10 hyperparameter sets on GBDT to test the 10 splitted datasets. Then obtained the average of seven metrics on the 10 splitted datasets. The result shows the second parameters set obtains the best performance in all metrics. And we make the second parameters set as the optimal parameters for GBDT with Bayesian. Similarly, the result shows the sixth parameters set obtains the best performance in all metrics. And we make the sixth parameters set as the optimal parameters for GBDT with Random.

**Supplementary datasheet 2.** The average value and Variance of the four methods. The results obtained on the 10 splitted datasets. The average shows the proposed method perform best in all metrics. But Variance result shows the proposed method perform stably in the accuracy, balance accuracy, and F1 score. And other metrics perform relatively stable compared with other methods.
